# Monitoring Seasonal Changes in Winery-Resident Microbiota

**DOI:** 10.1371/journal.pone.0066437

**Published:** 2013-06-19

**Authors:** Nicholas A. Bokulich, Moe Ohta, Paul M. Richardson, David A. Mills

**Affiliations:** 1 Department of Viticulture and Enology, University of California Davis, Davis, California, United States of America; 2 Department of Food Science and Technology, University of California Davis, Davis, California, United States of America; 3 Foods for Health Institute, University of California Davis, Davis, California, United States of America; 4 MicroTrek Inc., San Francisco, California, United States of America; Missouri University of Science and Technology, United States of America

## Abstract

During the transformation of grapes to wine, wine fermentations are exposed to a large area of specialized equipment surfaces within wineries, which may serve as important reservoirs for two-way transfer of microbes between fermentations. However, the role of winery environments in shaping the microbiota of wine fermentations and vectoring wine spoilage organisms is poorly understood at the systems level. Microbial communities inhabiting all major equipment and surfaces in a pilot-scale winery were surveyed over the course of a single harvest to track the appearance of equipment microbiota before, during, and after grape harvest. Results demonstrate that under normal cleaning conditions winery surfaces harbor seasonally fluctuating populations of bacteria and fungi. Surface microbial communities were dependent on the production context at each site, shaped by technological practices, processing stage, and season. During harvest, grape- and fermentation-associated organisms populated most winery surfaces, acting as potential reservoirs for microbial transfer between fermentations. These surfaces harbored large populations of *Saccharomyces cerevisiae* and other yeasts prior to harvest, potentially serving as an important vector of these yeasts in wine fermentations. However, the majority of the surface communities before and after harvest comprised organisms with no known link to wine fermentations and a near-absence of spoilage-related organisms, suggesting that winery surfaces do not overtly vector wine spoilage microbes under normal operating conditions.

## Introduction

Food fermentations occur under conditions in which microbial activities–from both intentionally inoculated and environmental organisms–are an inherent part of the process, playing important roles in determining product quality characteristics, as well as promoting spoilage [1]. Mounting evidence points to the involvement of indigenous, processing facility-associated microbes in the fermentations of wine [2], [3], beer [4], and cheese [5], [6]. The overarching goal of processing decisions for all of these fermentations is the management of these beneficial microbial ecosystems within the production environment. This is reflected in the adoption of traditional fermentation practices that modulate the incumbent microbiota, including: temperature control, oxygen limitation, adjunct ingredients, and cleaning procedures. However, the interplay between fermentation communities and processing environments remains poorly understood, and studies of microbial trafficking within food and beverage fermentation facilities are limited.

Recent advances in high-throughput, short-amplicon sequencing (HTS) technologies have revolutionized the study of microbial communities inhabiting diverse environments, enabling comprehensive microbial surveys with detection sensitivities previously untenable using culture-based techniques. One target facilitated by this innovation has been the microbial communities present in indoor environments including hospitals [7], [8], office spaces [9], public restrooms [10], and domestic kitchens [11]. Most studies of the built environment, however, have focused on potential pathogens in human-inhabited indoor spaces. No studies to date have used HTS methods to comprehensively profile the microbial communities of a food processing facility, where, as in the case of wine and other fermentations, microbial communities play a tripartite role in determining: 1) the chemosensory qualities of the final product; 2) product spoilage; and 3) the healthfulness of the product for human consumption. Consequently, surveillance of food and beverage fermentation facilities–and more generally food processing facilities–is paramount to understanding the role environmental microbiota play in shaping holistic product qualities.

Winemaking is a seasonal, agricultural practice employing traditional production techniques, dedicated facilities, and specialized equipment. Production schedules revolve around grape harvest, occurring during a brief period in the autumn, but the winery is active year-round, conducting wine to its final resting place in the wine bottle. Grapes are transported to the winery, crushed, pressed, fermented, and aged–traditionally in oak barrels–prior to bottling. Each of these stages involves a set of specialized equipment for handling the product in its gradual transition from grapes to wine, and occurs in dedicated areas of the winery. These processing areas are often maintained under temperature/environmental conditioning with the explicit objective of managing microbial activity during fermentation and maturation. During this journey, the fermenting wine contacts many different surfaces and is exposed to many different environments, all potential episodes for bidirectional microbial exchange.

The impact of a select number of bacteria and fungi on wine quality is well established, including both beneficial and detrimental roles [12], [13], [14]. Aside from the inoculation of *Saccharomyces* yeasts to initiate fermentation, several other yeast species are recognized for their role in fermentation. Non-inoculated, “wild” yeasts are popularly considered to enhance the “complexity” of wine fermentation through the production of a broader spectrum of sensory-active compounds than that produced from a pure inoculum alone [15]. *Oenococcus oeni* or other lactic acid bacterial species (LAB) are also often inoculated to perform a secondary, malolactic fermentation, but otherwise the role of bacteria in wine fermentation is generally considered detrimental [12]. A number of wild yeasts and bacteria are considered spoilage organisms in wine fermentations, decreasing final quality through the production of off-flavors, hazes, carbonation, or other defects [13], [14].

The origins of most microbes in wine fermentations are poorly understood and generally assumed to be from grapes [16]. However, the primary microbes involved both positively and negatively in wine fermentations–including *Saccharomyces*, *Brettanomyces,* and *Oenococcus oeni*–are only detected as minor populations on the surface of healthy grapes, if at all [16], [17], [18]. Another prevailing source for the transfer of these microbes between fermentations is the winery environment itself [3]. Winery equipment–including crush/press equipment and barrels–often involves difficult to clean, porous surfaces. Bathed with the nutritious medium of grape juice on a seasonal basis, these surfaces become very promising sites for microbial adsorption and biofilm production, potentially leading to continuous shedding of microbes into successive batches of wine. Specific *Saccharomyces* strains can become established on winery surfaces, resulting in repeatable detection over multiple years in uninoculated wines [19], [20], [21], [22]. The distribution of non-*Saccharomyces* fungi on specific winery surfaces has been studied to a limited extent at isolated timepoints using culture-based techniques [2], [3], [23]. Airborne populations of LAB [24], non-*Saccharomyces* yeasts [25], and molds [26], [27], [28] have also been investigated in wineries using culture-based techniques. However, the source and trafficking of indigenous microbes in wine fermentations remains a highly contentious topic, particularly of spoilage-related organisms [16], [17], [18], and all previous studies of winery surface microbiota have relied on culture-based techniques, which are prone to biases for studying food fermentation microbiota [1], [12]. Moreover, most studies of winery environments have focused on microorganisms previously cultured from wine fermentations. The complete temporospatial ecology within wineries is largely unknown, and warrants investigation given the role many uninoculated species play in shaping wine quality characteristics–as well as wine spoilage.

To elucidate the microbial landscape of winery surfaces during harvest, surface swab surveillance of the winery at the University of California-Davis Department of Viticulture and Enology was conducted across the course of a single harvest vintage. A HTS approach was used to monitor the bacterial and fungal communities of prominent winery surfaces and equipment that encounter grapes/wine at various stages of wine production, mapping the transitions in these communities across time and space. Results demonstrate that the microbial communities inhabiting winery surfaces fluctuate over the course of harvest, but retain semi-stable core patterns throughout this period. Furthermore, these communities exhibit spatial diversification reflecting the functional applications of each winery surface, but there was no evidence of conditions promoting the establishment of spoilage-related organisms. Importantly, *Saccharomyces cerevisiae* and other beneficial fermentation-related yeasts were detected on winery surfaces prior to harvest, indicating that establishment of these organisms may play a role in populating early wine fermentation microbiota.

## Materials and Methods

### Facility Description

**Figure 1:**
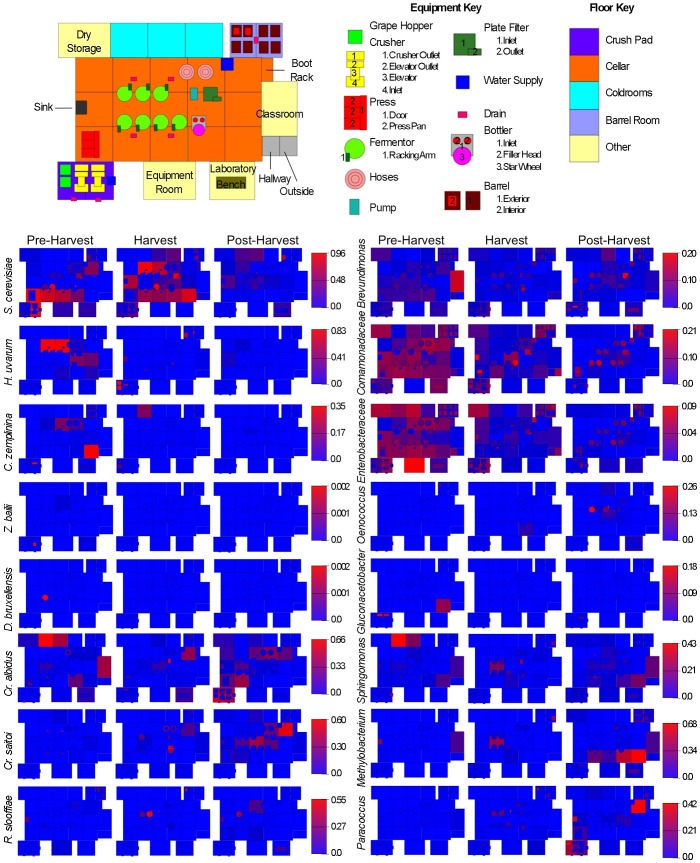
Spatial distribution heatmaps of yeasts and bacteria in winery environment across harvest. Plots indicate relative abundance of yeast (left) and bacterial taxa (right) detected by short-amplicon HTS reads across winery surfaces at different stages relative to harvest. Scales on right represent relative abundance scale (maximum 1.0) for each row of plots.

All samples were collected from the Robert Mondavi Institute for Food and Wine Science Winery (University of California, Davis). This pilot-scale winery employs three full-time staff and processes 58 tons of grapes (corresponding to 329 hl of wine) per annum. These consist primarily of the grape varieties Cabernet Sauvignon, Chardonnay, Grenache, Merlot, Barbera, and Zinfandel. As it is a teaching winery, this facility encounters more human traffic than normal for a winery of its size, but otherwise operates as a fully functional winery with year-round operations. In addition, student projects involving intentional inoculation of non-*Saccharomyces* yeasts occur in small-scale fermentations located in the cold rooms (Fig. 1). Otherwise, only common wine fermentation inocula, *Saccharomyces cerevisiae*, *Saccharomyces bayanus*, and *Oenococcus oeni* are added to wines and normal production methods are observed.

The winery was built in 2010 and has been in continuous operation since. All equipment is state-of-the-art, and fermentation vessels are purpose-built, stainless steel tanks. All equipment is cleaned immediately after use, using only water, and sanitized with a peroxy-acetic acid solution immediately prior to use. In addition, a 1% solution of a non-chlorinated, KOH-based cleaning agent is used for tartrate removal from fermentation equipment as necessary. The water used for cleaning is carbon-filtered, non-chlorinated water drawn from an underground reservoir. As equipment was swabbed as is in this study, most samples represent cleaned but non-sanitized surfaces.

Samples were collected at three separate time points: before any grapes were harvested (August 3, 2012), in the middle of harvest (October 3, 2012), and after the completion of harvest (December 12, 2012). The microbial communities of winery surfaces can be expected to depend heavily on the design and processing activities occurring in a given facility at a given time, and the following production transactions occurred on or just prior to the sampling dates. At the pre-harvest time-point (1 day prior to start of harvest), equipment was positioned for harvest and was last cleaned before putting away at the end of harvest 2011, but unsanitized. Small batches of wine were sterile-filtered and bottled, totaling 232.5 L, and 2,158 L of wine were dumped directly into the main cellar drains (as a teaching winery, only small volumes are packaged for educational purposes), but otherwise most of the facility was clean and dormant. On the day samples were collected at peak harvest (October 3, 2012), a tank of Cabernet Sauvignon and a tank of Merlot were drained and pressed (these were inoculated with *S. cerevisiae* previously), and *S. cerevisiae* was added to a tank of Barbera grape must to initiate fermentation. The week before this period consisted of similar activities, crushing, inoculating, and pressing lots of Merlot, Grenache, and Zinfandel. During the post-harvest sampling time, no activities occurred in the winery, and the only transactions occurring in the winery for the previous week were racking and filtering finished Cabernet Sauvignon fermentations.

### Sample Collection and DNA Extraction

Surfaces were sampled with sterile cotton-tipped swabs (Puritan Medical, Guilford, ME). Swabs were moistened with sterile phosphate-buffered saline and streaked across a 10 cm square area (or equivalent) of the target surface in two perpendicular series of firm, overlapping S-strokes, rotating the swab to ensure full contact of all parts of the swab tip and the surface. Swab tips were snapped off into sterile 1.5 mL polyethylene tubes against the inner edge of the tube without manual contact. Samples were placed on ice and frozen immediately in a –20°C freezer for storage. The cotton tip of each swab was aseptically removed from the shaft and placed directly into the 96-well lysis plate provided in the ZR-96 Fecal DNA extraction kit (Zymo Research, Irvine, CA). DNA was extracted using the standard protocol for the ZR-96 kit, with bead beating with a Genogrinder high-throughput tissue homogenizer (SPEX SamplePrep, Metuchen, NJ) and stored at –20°C until further processing. A complete list of each sample collected is presented in Tables S1, S2 [29].

### Sequencing Library Construction

Amplification and sequencing were performed as described previously for bacterial [30] and fungal communities [31]. Briefly, the V4 domain of bacterial 16S rRNA genes was amplified using primers F515 (5′-*NNNNNNNN* **GT**GTGCCAGCMGCCGCGGTAA-3′) and R806 (5′-GGACTACHVGGGTWTCTAAT-3′) [32], with the forward primer modified to contain a unique 8 nt barcode (italicized poly-N section of primer above) and 2 nt linker sequence (bold, underlined portion) at the 5′ terminus. All F515 primer barcodes used are presented in Table S1. PCR reactions contained 5–100 ng DNA template, 1X GoTaq Green Master Mix (Promega, Madison, WI), 1 mM MgCl_2_, and 2 pmol of each primer. Reaction conditions consisted of an initial 94°C for 3 min followed by 35 cycles of 94°C for 45 s, 50°C for 60 s, and 72°C for 90 s, and a final extension of 72°C for 10 min. Fungal internal transcribed spacer (ITS) 1 loci were amplified with primers BITS (5′-*NNNNNNNN* **CT**ACCTGCGGARGGATCA-3′) and B58S3 (5′-GAGATCCRTTGYTRAAAGTT-3′) [31], with a unique 8 nt barcode and linker sequence incorporated in each forward primer. All BITS primer barcodes used are presented in Table S2. PCR reactions contained 5–100 ng DNA template, 1X GoTaq Green Master Mix (Promega), 1 mM MgCl_2_, and 2 pmol of each primer. Reaction conditions consisted of an initial 95°C for 2 min followed by 40 cycles of 95°C for 30 s, 55°C for 30 s, and 72°C for 60 s, and a final extension of 72°C for 5 min. Amplicons were combined into two separate pooled samples (keeping bacterial and fungal amplicons separate) at roughly equal amplification intensity ratios, purified using the Qiaquick spin kit (Qiagen), and submitted to the UC Davis Genome Center DNA Technologies Core for Illumina paired-end library preparation, cluster generation, and 250 bp paired-end sequencing on an Illumina MiSeq instrument in two separate runs.

### Data Analysis

Raw and filtered sequence counts are summarized in Table S3. Raw fastq files were demultiplexed, quality-filtered, and analyzed using QIIME 1.5.0 [29]. The 250-bp reads were truncated at any site of more than three sequential bases receiving a quality score<Q20, and any read containing ambiguous base calls or barcode/primer errors were discarded, as were reads with <75% (of total read length) consecutive high-quality base calls [33]. For ITS sequences, primer sequences were trimmed from the ends of each sequence and operational taxonomic units (OTUs) were clustered *de novo* using the QIIME implementation of UCLUST [34], with a threshold of 97% pairwise identity. Bacterial 16S sequences were clustered using the QIIME subsampled reference OTU-picking pipeline using UCLUST-ref [34] against the Greengenes 16S rRNA database (February 2011 release) [35], clustered at 97% pairwise identity. OTUs were classified taxonomically using a QIIME-based wrapper of BLAST [36] against the Greengenes 16S rRNA database (for 16S sequences) or the UNITE [37], [38] database (for ITS sequences) modified as described previously [31]. Any OTU comprising less than 0.001% of total sequences for each run were removed prior to further analysis, calibrating against a known mock community in the ITS sequencing run [33]. Bacterial 16S sequences were aligned using PyNAST [39] against a reference alignment of the Greengenes core set [35]. From this alignment, chimeric sequences were identified and removed using ChimeraSlayer [40] and a phylogenetic tree was generated from the filtered alignment using FastTree [41]. Sequences failing alignment or identified as chimera were removed prior to downstream analysis.

Jackknifed beta diversity estimates (between-sample diversity comparisons) were calculated within QIIME using weighted and unweighted UniFrac [42] distance between samples for bacterial 16S rRNA reads (evenly sampled at 450 reads per sample) and Bray-Curtis dissimilarity for fungal ITS reads (evenly sampled at 400 reads per sample), subsampled 10 times without replacement. From these estimates, principal coordinates were computed to compress dimensionality into two-dimensional principal coordinate analysis (PCoA) plots. In order to determine whether sample classifications (sample time, equipment type, location) contained differences in OTU diversity, permutational MANOVA [43] with 999 permutations was used to test the null hypothesis that sample groups were not statistically significant based on evenly sampled UniFrac and Bray-Curtis distance matrices, using the QIIME-wrapped R module Adonis. For all classifications rejecting this null hypothesis, one-way ANOVA was used to determine which taxa differed significantly (with Bonferroni error correction) between sample groups.

Alpha diversity estimates (within-sample diversity) were calculated within QIIME using Shannon entropy and Phylogenetic diversity (PD whole tree) [44]. OTU tables were rarefied with 10 permutations, and alpha diversity statistics calculated at even sampling depths. Two-sample T-tests were used to test whether significant differences exist between these scores for each sample classification (surface type, sampling time) at a single sampling depth (400 OTUs).

Environmental surveillance heatmaps were generated based on taxonomic abundance tables generated in QIIME and visualized using SitePainter 1.1 [45].

### Quantitative PCR

In order to quantify net microbial biomass on winery surfaces throughout harvest season, quantitative PCR (QPCR) was used to enumerate total fungi and bacteria. Surfaces that directly contact grapes and grape must–and thus have the greatest impact on early fermentation communities–were selected for analysis, consisting of crush equipment, press, and fermentor samples. QPCR was performed in 20-µL reactions containing 2 µL of DNA template, 5 pmol of each respective primer, and 10 µL of Takara SYBR 2X Perfect Real Time Master Mix (Takara Bio Inc). For quantification of total fungi, the primers YEASTF (5′-GAGTCGAGTTGTTTGGGAATGC-3′) and YEASTR (5′-TCTCTTTCCAAAGTTCTTTTCATCTT-3′) were used [46]. Reaction conditions involved an initial step at 95°C for 10 min, followed by 40 cycles of 15 s at 95°C, 1 min at 60°C, and 30 s at 72°C. For amplification of total bacteria, the primers Uni334F (5′-ACTCCTACGGGAGGCAGCAGT-3′) and Uni514R (5′-ATTACCGCGGCTGCTGGC-3′) [47] were used. Reaction conditions consisted of an initial hold at 95°C for 20 s, followed by 40 cycles of 4 s at 95°C and 25 s at 65.5°C. All reactions were performed in triplicate in optical-grade 96-well plates on an ABI Prism 7500 Fast Real-Time PCR System (Applied Biosystems). The instrument automatically calculated cycle threshold (*C_T_*), efficiency (*E*), confidence intervals, and cell concentration (fungi) or 16S rRNA gene copy number (bacteria) by comparing sample threshold values (*C_T_*) to a standard curve of serially diluted genomic DNA extracted from a known concentration of *Saccharomyces cerevisiae* cells or *Escherichia coli* genome copies. Two-sample T-tests (with even or uneven sample sizes, as appropriate) were calculated to test significant differences between individual sample classifications.

## Results and Discussion

### Winery Surveillance across Harvest

Wine fermentations contain a wealth of microbial diversity [30] originating from two fertile sources: grapes and the winery environment. During the process of wine fermentation, grape must and ensuing wine encounters a large area of functional surfaces under many different operating conditions. However, while some efforts have been made to characterize grape microbiota [13], [16], [17], [18], [48], the microbial consortia of winery surfaces has only been described to a limited extent on specific surfaces [2], [3], [19]–[28]. Thus, our initial goal was to describe the microbial landscape of a winery to elucidate what microbial reservoirs exist within a winery and better understand how wine fermentations interact with this environment. Samples were collected at three separate time points during the 2012 vintage: before any grapes were harvested (“pre-harvest”, August 3, 2012), in the middle of harvest (“harvest”, October 3, 2012), and after the completion of harvest (“post-harvest”, December 12, 2012). At the pre-harvest time-point (1 day prior to start of harvest), equipment was positioned for harvest and was last cleaned before putting away at the end of harvest 2011, but unsanitized.

The winery microbiome changes across both time and space, reflecting both the seasonality of the process and the functional specialization of different equipment and surfaces within the winery (Fig. 1, Fig. S1). Before bringing grapes into the winery, winery surfaces were dominated by aerobic, non-fermentation-related bacteria, primarily *Pseudomonas, Comamonadaceae, Flavobacterium, Enterobacteraceae, Brevundimonas,* and *Bacillus*. The fungal communities of these surfaces were–importantly–largely comprised of *Saccharomyces cerevisiae* (as much as 96% relative abundance) and other fermentative yeasts, principally *Hanseniaspora uvarum*, as well as *Cryptococcus* spp. and molds including *Aureobasidium pullulans* and *Aspergillus* spp. Crush equipment and barrel room surfaces were the primary residences of these molds (Fig. S1). The pre-harvest communities represent the resting state of the winery, but are more importantly the first populations encountered by fresh grape juice prior to fermentation, so the composition of this community can crucially impact wine fermentation qualities downstream. Colonization of winery surfaces by *Saccharomyces* has been reported previously and is probably an important source of this yeast in wine fermentations, particularly in non-inoculated wines [19], . However, none of these studies quantitatively demonstrated *Saccharomyces* as an abundant, dominant pre-harvest population on winery surfaces. *Hanseniaspora* has also been previously reported to colonize winery surfaces [3] and plays an important role in the early stages of wine fermentation [13]. While this yeast is typically present on grapes [17], winery surface establishment may ensure that the same strains are introduced to successive batches and vintages of wine as previously shown for *Saccharomyces* [19], [20], [21], possibly supporting the reproducibility, as well as regionality, of wine sensory characteristics produced at a given facility. Another yeast detected at lower levels on winery surfaces prior to harvest was *Candida zemplinina* (Fig. 1). Like *Hanseniaspora*, this fructophilic yeast [49], [50] is gaining recognition as an important player in some wine fermentations [51], and once it gains entry to the winery, surface establishment may provide repeated inoculation in successive batches, helping shape the sensory characteristics of the wine. However, no previous studies have detected this yeast on winery surfaces. Non-*Saccharomyces* yeasts are important members of wine fermentations and increase the “complexity” of wine aroma through the production of a greater range of sensory-active compounds than that produced by *Saccharomyces* alone [15]. Many are detected on grapes [17] but their ecological dispersion throughout a winery is not well established. Establishment of different yeast species carried in from the vineyard may populate the resident microbiota of a given winery, potentially shaping the regional, signature sensory characteristics of those wines vintage-to-vintage.

**Figure 2:**
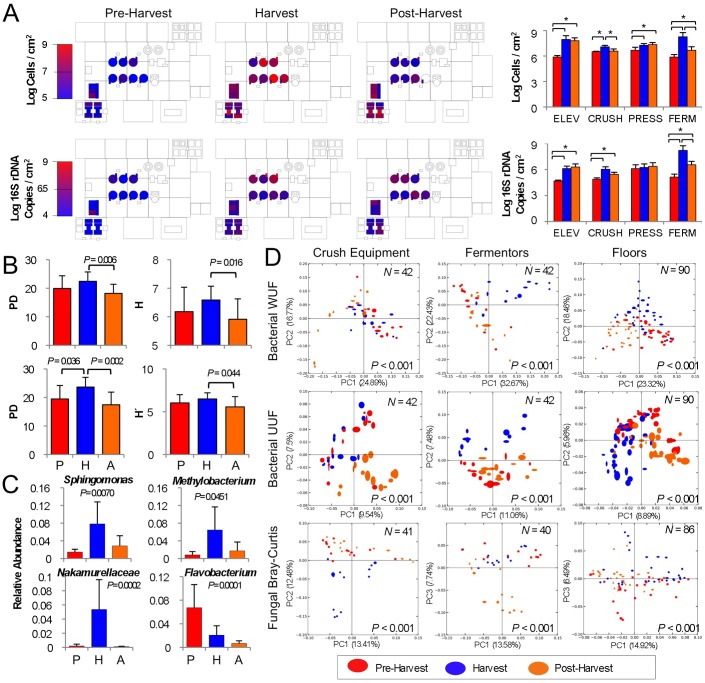
Seasonal flux in species diversity observed across winery surfaces.s (A) Absolute abundance of fungi (top, as cells/cm^2^) and bacteria (bottom, as 16S rRNA gene copies/cm^2^) detected on select surfaces by QPCR at different stages relative to harvest. Bar plots to right indicate mean (±SD) abundance of all grape elevator (ELEV), crusher (CRUSH), press, and fermentor (FERM) communities before (red), during (blue), and after harvest (orange). *P<0.05, two-sample T-tests. (B) Bacterial phylogenetic diversity (PD), a measurement of net branch-length distance on a single phylogenetic tree that is covered in each sample (left) and bacterial Shannon entropy (right) average (±SD) alpha-diversity scores for grape crush-related equipment (top, *N* = 42) and floor samples (bottom, *N* = 90). Two-sample T-test *P* scores shown for significantly differing categories. (C) Average relative abundance (maximum 1.0) ±SD of select bacterial genera associated with fermentation vessel samples at peak harvest. One-way ANOVA *P* values (with Bonferroni error correction) shown for significance between each category. *P*, pre-harvest (*N* = 14); *H*, harvest (*N* = 14); *A*, post-harvest (*N* = 14). (D) Jackknifed beta-diversity PCoA plots for crush equipment (left), fermentation vessels (center), and floor surface samples (right) categorized by sampling date. Value in lower-right corner indicates permutational MANOVA *P*-value between categories, sample size (*N*) in upper-right corner. *UUF*, unweighted UniFrac distance; *WUF*, abundance-weighted UniFrac distance.

When harvest begins, the winery becomes inundated with grapes and fermenting grape juice, so it is no surprise that *Saccharomyces* became more widespread in the environment, especially around fermentation tanks (Fig. 1). More startling, however, was the low relative abundance of other fermentation-related microbiota detected during this stage. *Hanseniaspora* and *C. zemplinina*, in addition to other fermentative yeasts (e.g., *Lachancea [formerly Kluyveromyces] thermotolerans* and *Torulaspora delbrueckii*), were still detected at low levels across the winery, and did not change significantly from pre-harvest levels. However, absolute abundance of both fungal and bacterial communities increased significantly on all grape processing equipment (grape elevator, crusher, press) and fermentor surfaces compared to pre-harvest levels, except for press-associated bacterial communities (Fig. 2A). Thus, most of these populations exhibited modest increases in absolute abundance on these surfaces. In particular, grape elevator and fermentor surfaces saw a 100-fold increase in mean bacterial and fungal abundance (Fig. 2A), indicating that grape contact introduced a bolus of microbial biomass into the winery and stimulated growth of select communities on these surfaces. Although the winery was awash with grape juice at this period, common fermentation-related and spoilage organisms (e.g., *Dekkera* and *Zygosaccharomyces* yeasts; *Acetobacteraceae* and *Lactobacillaceae* bacteria) were not detected or were only detected sporadically or at very low levels (Fig. 1). Instead, non-equipment surfaces saw the growth of molds (especially *Wallemia* spp., Fig. S1) and a significant increase in bacterial alpha diversity (within-sample species diversity) (Fig. 2B), indicating that community abundance became spread over a greater number of species and became more phylogenetically diverse. Fermentor and fermentation-related surfaces developed significant populations of *Sphingomonas* (7.8±4.9% relative abundance), *Methylobacterium* (6.4±5.2%), and *Nakamurellaceae* (5.3±4.2%) among bacteria (Fig. 2C), and the yeasts *Cryptococcus saitoi* and *Rhodotorula* spp. became more abundant throughout the winery (Fig. 1). *Sphingomonas* and *Methylobacterium* have both been detected in wine fermentations previously [30] and contact with winery surfaces may explain the detection of these species, which are otherwise unknown in wine fermentations. These shifts in both surface types also resulted in significant shifts in beta diversity (between-sample diversity comparison) clustering patterns (Fig. 2D), indicating broad community shifts at harvest compared to pre- and post-harvest sampling times. Crush equipment bacterial communities displayed more subtle and sporadic changes that did not result in significant shifts in species diversity, so no change in beta diversity clustering patterns was observed (Fig. 2D). However, fungal communities observed at peak harvest exhibited significant shifts in fungal beta diversity (Fig. 2D), marked by increased detection of molds (e.g., *Wallemia*, *Alternaria*, *Aspergillus*, *Penicillium*) (Fig. S1) and yeasts (*Hanseniaspora*, *Wickerhamomyces, Cryptococcus*) (Fig. 1) typically associated with grape surfaces [17].

After harvest, winemaking activities continue, as the wine continues to ferment and then age for several more months. However, grapes, which are volumetrically the primary input of microbial biomass to the winery, are no longer present and most crush equipment is cleaned and put away for the vintage. At this stage, winery surfaces did not entirely return to their original microbial composition, but instead microbial changes continued around the fermentors and barrels, which were being emptied and filled during this period (Fig. 1). Absolute quantities of the fungal and bacterial communities inhabiting fermentor surfaces declined significantly compared to peak-harvest levels, as did fungal communities on the grape crusher (Fig. 2A). Alpha diversity of floors and crush equipment also decreased significantly compared to harvest-period levels (Fig. 2B) and fermentor samples returned to their pre-harvest bacterial beta diversity cluster (Fig. 2D). However, changes continued to occur in both fungal and bacterial communities elsewhere in the winery, manifested in beta diversity shifts in the bacterial communities of floor samples and crush equipment and fungal communities of fermentors (Fig. 2D). Populations of *Methylobacterium, Sphingomonas*, and *Paracoccus* continued to increase on non-fermentor surfaces throughout the winery, as did the yeasts *Cryptococcus* and *Rhodotorula* (Fig. 1). In some respects, the pre-harvest *status quo* was restored (e.g., the re-establishment of fermentor bacterial communities and decrease of microbial biomass on grape processing and fermentor surfaces). In other respects it is obvious that this was another stage in a more complicated seasonal succession, and it is unclear whether shifts at this stage were conditioned by anthropogenic factors (e.g., harvest residues, fermentation run-off) or environmental factors (e.g., the dramatic decrease in ambient temperature and increase in humidity post-harvest compared to earlier time periods).

### Spatial Variation in Surface Microbiota

Each item of equipment in a winery is specially designed for a defined purpose, and under normal operating scenarios encounters chemically similar solutions under predictable physical conditions. For example, grape hoppers will carry only grapes in various forms; crush equipment will be bathed in concentrated sugar (grape juice) on a seasonal basis; fermentors will house fermenting grapes/juice under anaerobiosis and gradually increasing ethanol concentrations; barrels will hold finished wine (except in the case of barrel fermentations); floors will be spattered with grape juice and wine. Accordingly, it may be expected that each surface will develop its own niche environment for microbial specialization. In a winery environment, this adaptation process becomes particularly important, as enrichment of spoilage organisms under certain conditions represents a threat to wine quality, and identifying these sites can become critical for improved fermentation management.

**Figure 3:**
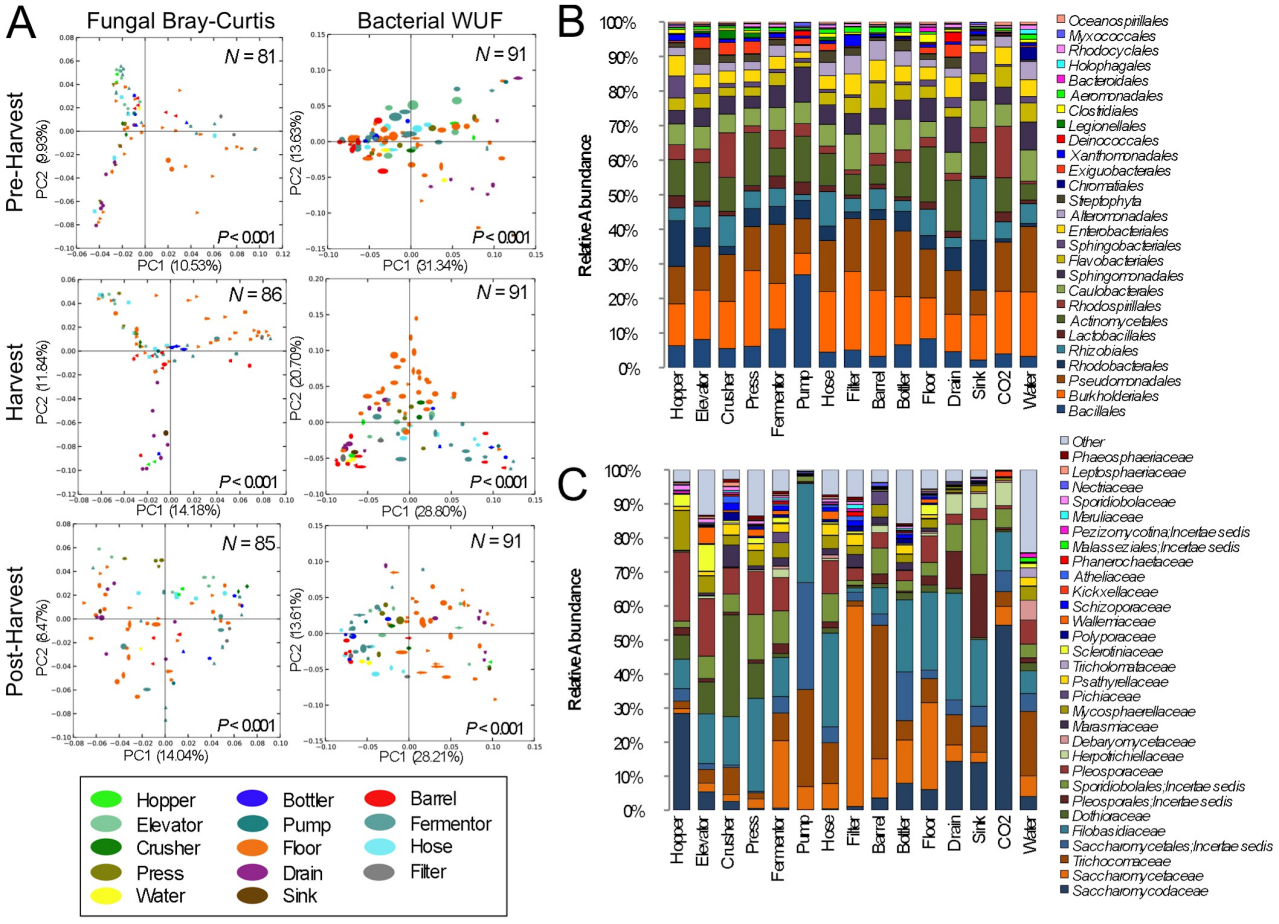
Winery surface species diversity illustrates functional niche selection. (A) Jackknifed beta-diversity PCoA plots for pre-harvest (top), peak harvest (center), and post-harvest (bottom) samples categorized by surface type. Values in lower-right corners indicate permutational MANOVA *P*-values between categories, sample size (*N*) in upper-right corners. *WUF*, abundance-weighted UniFrac distance. Relative taxonomic distribution of (B) order-level bacterial community abundance and (C) family-level fungal community abundance of all surface type categories. Each column represents average abundance of microbial taxa detected in all samples from each category for all three timepoints.

**Figure 4:**
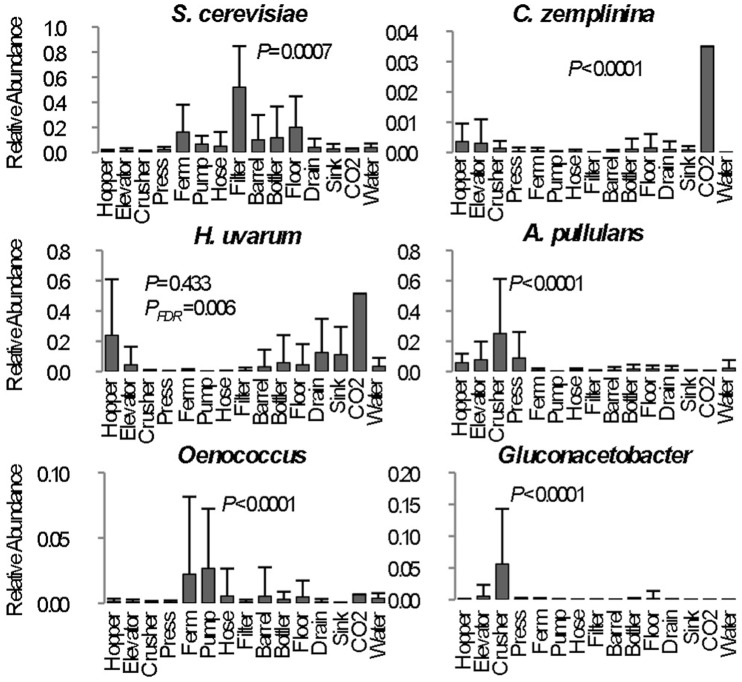
Significant between-category differences in abundance of fermentation-related taxa reflects niche selection within winery surface types. Each column represents average relative abundance (maximum 1.0) ± SD of select microbial taxa detected in all samples from each category for all three timepoints. One-way ANOVA *P* values (with Bonferroni error correction) shown for significance between each category. *P_FDR_ = *false discovery rate*-corrected P* value; *Ferm*, fermentor sample mean. Only one sample was collected for CO_2_ tube category and thus not included in statistical calculations.

**Figure 5:**
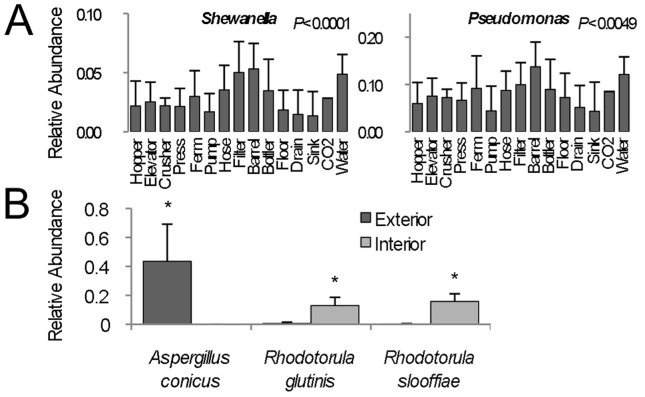
Barrel surfaces comprise unique microbial communities. (A) Average relative abundance (maximum 1.0) ± SD of *Shewanella* (left) and *Pseudomonas* (right) detected in all samples from each category for all three timepoints. One-way ANOVA *P* values (with Bonferroni error correction) shown for significance between each category. (B) Average relative abundance (±SD) of fungal species exhibiting significant differences between exterior (dark grey, *N = *5) and interior (light grey, *N* = 3) barrel surfaces prior to harvest. *P<0.05, two-sample T-test.

Different winery surfaces show clear evidence of niche specialization, but each ecosystem is subject to seasonal flux due to harvest disruption (Fig. 3). Crush equipment (hopper, elevator, crusher, and press samples) clustered together at each stage (Fig. 3A), with varying degrees of cluster tightness, due to the common medium encountered by these items: grapes. Thus, these surfaces were all populated by similar molds (e.g., *Aureobasidium pullulans*), yeasts (*Hanseniaspora, Candida*), and bacteria (*Acetobacteraceae*) associated with grapes, distinguishing them from most other environments (Fig. 1, Fig. 3B, Fig. 3C, Fig. 4, Fig. S1). Likewise, fermentation equipment samples (fermentors, hoses, filters, and pumps) tended to cluster together (with a certain degree of spread, especially for fungal communities), and somewhat near the crush equipment (Fig. 3A). This may reflect that all deal strictly with fermenting and fermented wine, hence displaying significantly higher populations of *S. cerevisiae* and *Oenococcus* (Fig. 4). Both these groups clustered closely with barrels and bottlers before and after harvest, but drifted during harvest, as the influx of viticultural material and active fermentation introduced the development of distinct microbial communities (Fig. 3A). The bacterial communities of barrel surfaces were similar to those elsewhere in the winery (Fig. 3B), dominated by *Pseudomonas*, *Comamonadaceae*, *Brevundimonas*, and *Flavobacterium*, but with significantly higher populations of *Pseudomonas* and *Shewanella* compared to most other surfaces (Fig. 5A). The fungal communities of barrels were distinguished by higher abundances of the filamentous fungi *Aspergillus conicus* and *Aspergillus restrictus*, compared to other surface types (Fig. S1). Moreover, *A. conicus* was significantly higher on the outer surface of barrels, whereas the yeasts *Rhodotorula slooffiae* and *Rhodotorula gluntinis* were more abundant on inner surfaces prior to harvest (Fig. 5B). These are all oxidative fungal species with no known role in wine fermentations, though they are frequently detected in grape must [13]. Floor samples showed a high degree of beta-diversity spread, encompassing diverse environments and conditioned zones within the winery. Nevertheless, these clustered away from most other sample types (except drains), especially during and after harvest (Fig. 3A), as microbial communities at these sites became altered by contact with grape material during harvest.

Surprisingly, most known wine spoilage-related microbes were undetected or detected at very low levels across winery surfaces. With the exception of *Acetobacteraceae* on crush equipment (Fig. 4), none of the surface types could be identified as a significant niche environment for growth of any spoilage-related organisms (*P*>0.05). The near-absence of these spoilage organisms during harvest comes as a surprise, as many of these organisms, especially *Acetobacteraceae* and *Lactobacillaceae*, are commonly found on healthy, intact grape berries, and at much higher levels on damaged grapes [17], so would be expected to become dispersed more widely during harvest. Instead, most of the same non-fermentation-related species were detected on floors, barrels, and crush equipment over time at altered abundances, and spoilage-related species were detected only sporadically (Fig. 1). This may reflect the long-term viability of these organisms during periods of low nutrient availability (i.e., post-cleaning), under which the observed species may thrive due to alternative metabolic pathways not relying on grape-based substrates. Alternatively it may suggest that under proper sanitation conditions, non-fermentative resident communities dominate these functional niches, providing resistance against colonization by temporal spoilage organisms. Another unexpected finding was elevated detection of *H. uvarum* (51.5% relative abundance) and *C. zemplinina* (3.5% relative abundance) in a CO_2_ venting line attached to one fermentor (Fig. 4). This line is connected directly to the fermentor without a filter, and a sample was collected from the condensation collecting during active fermentation. Apparently, either backsplash or aerosols carried by CO_2_ emissions during active fermentation collect in these lines, harboring fermentation-related organisms, including a low level (0.24% relative abundance) of the spoilage yeast *Dekkera bruxellensis* in this sample. While this sample was only collected at a single timepoint, this finding highlights the potential for unique sites like this to serve as direct vectors for microbial transfer between fermentations. Regular cleaning should be employed in all winemaking scenarios to avoid establishment of spoilage organisms within otherwise benign microbial surface ecosystems.

### High-Density Microbial Surveillance

In this study, seasonal microbial surveillance in this winery detected temporal shifts associated with grape-associated communities introduced during harvest. However, the majority of these communities did not appear to establish on winery surfaces under normal cleaning conditions, and declined after harvest finished. An important exception is *S. cerevisiae* and *H. uvarum*, which appeared to colonize winery surfaces, a potential reservoir for introduction to early wine fermentation communities. It should be noted, however, that the microbial consortia of processing surfaces most likely depend on facility design, age, surface material, sanitation regimens, and processing decisions. Thus, these results cannot be generalized across all winemaking scenarios, as each winemaking facility may present certain unique conditions.

The fermentation of wine and other foods involves unprotected interaction with equipment surfaces and the processing facility environment at several stages, all opportunities for microbial exchange. Given the importance surface microbiota play in conducting aspects of these fermentations, routine facility surveillance may become a new approach for the study of fermentation microbiota in any food system. Under this new model, fermentations and the surrounding environment would be analyzed in tandem, recognizing that shifts in the microbial communities of either is not an independent phenomenon, and ambient changes may exert a far-reaching impact on product quality. In addition, high-density facility monitoring may become an important tool for improving sanitation and product quality in wineries and food processing facilities, where routine microbial surveillance can monitor communities at critical sites for improved process control.

## Supporting Information

Figure S1 Spatial distribution heatmaps of filamentous fungi in winery environment across harvest. Plots indicate relative abundance of filamentous fungi detected by short-amplicon HTS reads across winery surfaces at different stages relative to harvest. Scales on right represent relative abundance scale (out of 1.0 total) for each row of plots.
(TIFF)

Table S1 Sample Key and Barcode List for Bacterial Sequence Data.
(XLSX)

Table S2 Sample Key and Barcode List for Fungal Sequence Data.
(XLSX)

Table S3 Sequence Quality Filtering Counts.
(PDF)
