# Supplementary figures and images for: Monitoring Seasonal Changes in Winery-Resident Microbiota
Source: PLoS One. 2013 Jun 19;8(6):e66437. doi: 10.1371/journal.pone.0066437 (PMC3686677; doi:10.1371/journal.pone.0066437)

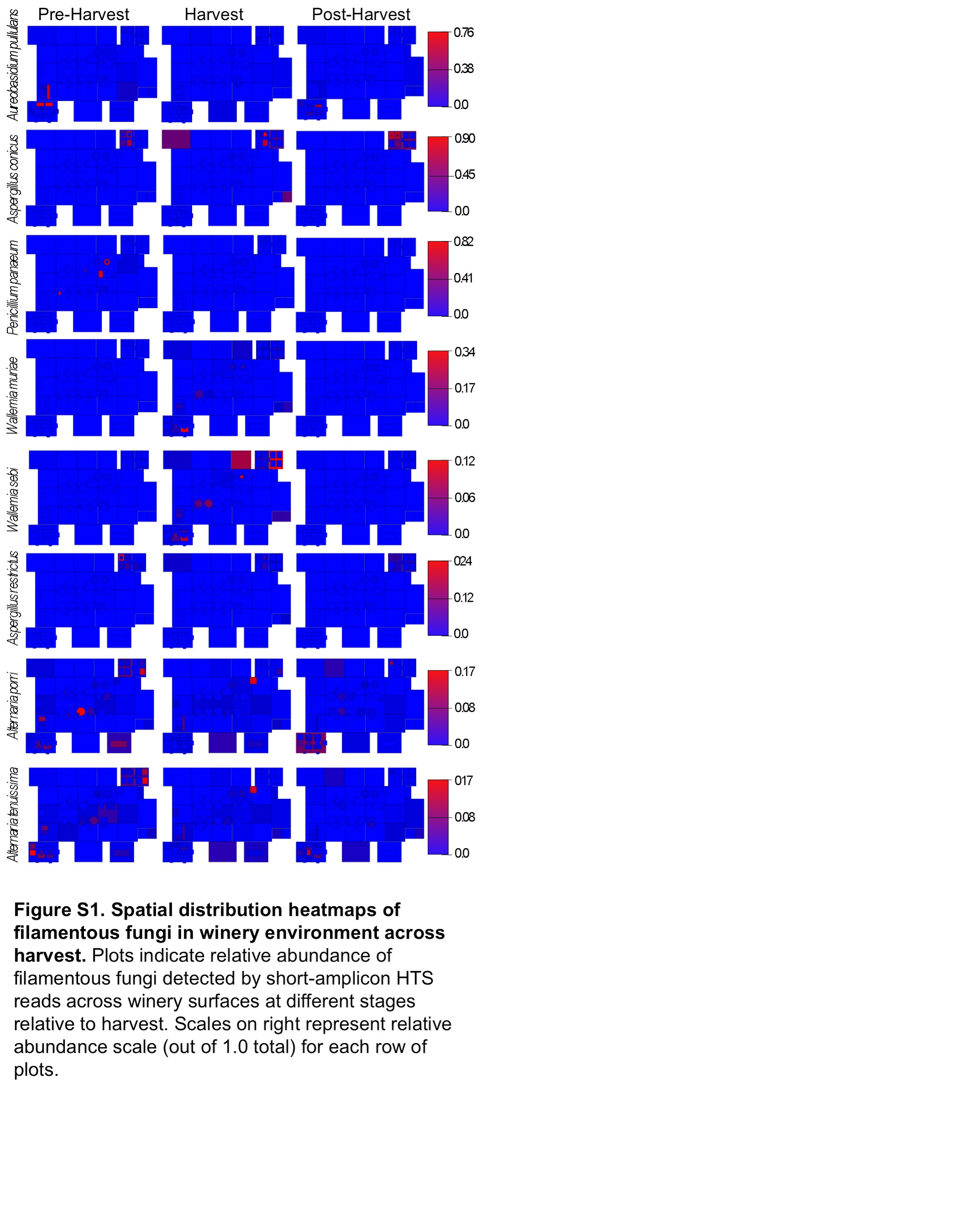

Supplement: Figure S1 — Spatial distribution heatmaps of filamentous fungi in winery environment across harvest. Plots indicate relative abundance of filamentous fungi detected by short-amplicon HTS reads across winery surfaces at different stages relative to harvest. Scales on right represent relative abundance scale (out of 1.0 total) for each row of plots. (TIFF) [file pone.0066437.s001.tiff]
