# Supplementary material for: Monitoring Seasonal Changes in Winery-Resident Microbiota
Source: PLoS One. 2013 Jun 19;8(6):e66437. doi: 10.1371/journal.pone.0066437 (PMC3686677; doi:10.1371/journal.pone.0066437)
Supplement: Table S3 — Sequence Quality Filtering Counts. (PDF) [file pone.0066437.s004.pdf]

Table S3. Sequence Quality Filtering Counts

	Bacteria	Fungi
Raw Sequences	27818526	27709944
Barcode Not in Mapping File[a]	22310626	24394275
Read Too Short After Quality Truncation	838602	656664
Count of N Characters Exceeds Limit	1743	48600
Demultiplexed Sequences	4667555	2610405
Chimeric Sequences	14418	-
Final Sequence Count	3810231	2542830

[a]Other samples in sequencing run account for high discard count
